# Supplementary material for: Viral metagenome characterization reveals species-specific virome profiles in Triatominae populations from the southern United States
Source: PLoS Negl Trop Dis. 2026 Feb 2;20(2):e0013576. doi: 10.1371/journal.pntd.0013576 (PMC12890172; doi:10.1371/journal.pntd.0013576)
Supplement: S1 Table — Summary of 23 Triatominae individuals with species, sex, geographic location, tissue type, QC, and T. cruzi detection. (PDF) [file pntd.0013576.s001.pdf]

Supplementary Table 1. Sample metadata and *T. cruzi* meta-assembly screening. Summary of 23 Triatominae individuals with species, sex, geographic location, tissue type, QC, and *T. cruzi* detection.

Individual	Sample ID	Tissue type	Stage/Sex	Sampling state	Sampling area	Location coordinates	Host species	QC	*T. cruzi* meta-assembly screening
**AZ1I6F**	AZ1I6FGUT	Gut	Female	Arizona	Desert Station	32.253846N, 111.089947W	*H. protracta*	PASS	non-infected
	AZ1I6FGONADS	Gonads	Female	Arizona	Desert Station	32.253846N, 111.089947W	*H. protracta*	PASS	non-infected
**AZ6I6M**	AZ6I6MGUT	Gut	Male	Arizona	Desert Station	32.2540685N, 111.0922009W	*H. protracta*	PASS	non-infected
	AZ6I6MGONADS	Gonads	Male	Arizona	Desert Station	32.2540685N, 111.0922009W	*H. protracta*	PASS	non-infected
**B40I6F**	B40I6FGUT	Gut	Female	Texas	Camp Bullis	29.7424452N, 98.5876910W	*T. indictiva*	PASS	non-infected
	B40I6FGONADS	Gonads	Female	Texas	Camp Bullis	29.7424452N, 98.5876910W	*T. indictiva*	PASS	non-infected
**B40I6M**	B40I6MGUT	Gut	Male	Texas	Camp Bullis	29.7424452N, 98.5876910W	*T. indictiva*	PASS	non-infected
	B40I6MGONADS	Gonads	Male	Texas	Camp Bullis	29.7424452N, 98.5876910W	*T. indictiva*	PASS	non-infected
**L14I6F**	L14I6FGUT	Gut	Female	Texas	Lackland Air Force Base	29.3767920N, 98.6849585W	*T. sanguisuga*	PASS	non-infected
	L14I6FGONADS	Gonads	Female	Texas	Lackland Air Force Base	29.3767920N, 98.6849585W	*T. sanguisuga*	PASS	non-infected
**L14I6M**	L14I6MGUT	Gut	Male	Texas	Lackland Air Force Base	29.3767920N, 98.6849585W	*T. sanguisuga*	PASS	non-infected
	L14I6MGONADS	Gonads	Male	Texas	Lackland Air Force Base	29.3767920N, 98.6849585W	*T. sanguisuga*	PASS	non-infected
**L14I6ME**	L14I6MEGUT	Gut	Male	Texas	Lackland Air Force Base	29.3767920N, 98.6849585W	*T. gerstaeckeri*	PASS	non-infected
	L14I6MEGONADS	Gonads	Male	Texas	Lackland Air Force Base	29.3767920N, 98.6849585W	*T. gerstaeckeri*	FAIL	non-infected
**L16I5**	L16I5GUT	Gut	L5	Texas	Lackland Air Force Base	29.3829725N, 98.6891581W	*T. gerstaeckeri*	PASS	infected
	L16I5GONADS	Gonads	L5	Texas	Lackland Air Force Base	29.3829725N, 98.6891581W	*T. gerstaeckeri*	PASS	non-infected
**NM9I6M**	NM9I6MGUT	Gut	Male	New Mexico	Hatch	32.6990360N, 107.1797240W	*T. rubida*	PASS	non-infected
	NM9I6MGONADS	Gonads	Male	New Mexico	Hatch	32.6990360N, 107.1797240W	*T. rubida*	PASS	non-infected
**L17I6F**	L17I6FGUT	Gut	Female	Texas	Lackland Air Force Base	29.3714983N, 98.6571148W	*T. gerstaeckeri*	PASS	non-infected
	L17I6FGONADS	Gonads	Female	Texas	Lackland Air Force Base	29.3714983N, 98.6571148W	*T. gerstaeckeri*	PASS	non-infected
**AZ4I5**	AZ4I5GUT	Gut	L5	Arizona	Desert Station	32.2540327N, 111.0917907W	*T. rubida*	PASS	non-infected
	AZ4I5GONADS	Gonads	L5	Arizona	Desert Station	32.2540327N, 111.0917907W	*T. rubida*	PASS	non-infected
**AZ1I6F**	AZ1I6FABD	Whole Abdomen	Female	Arizona	Desert Station	32.2575005N, 111.0842631W	*T. rubida*	PASS	infected
**L19I6F**	L19I6FABD	Whole Abdomen	Female	Texas	Lackland Air Force Base	29.3675352N, 98.6548306W	*T. gerstaeckeri*	PASS	non-infected
