# Supplementary material for: Viral metagenome characterization reveals species-specific virome profiles in Triatominae populations from the southern United States
Source: PLoS Negl Trop Dis. 2026 Feb 2;20(2):e0013576. doi: 10.1371/journal.pntd.0013576 (PMC12890172; doi:10.1371/journal.pntd.0013576)
Supplement: S2 Table — Twelve DNA isolates from gut samples analysed using vertebrate 12S rRNA gene sequences to identify blood meal sources. (PDF) [file pntd.0013576.s002.pdf]

Supplementary Table 2. Blood meal analysis of Triatominae gut tissues. Twelve DNA isolates from gut samples analysed using vertebrate 12S rRNA gene sequences to identify blood meal sources.

Sample name	Best blast hit acc.no.	Description	% identity	Length	e-value	note
**NM9I6MGUT**	DQ179667.1	*Neotoma albigula* isolate TK74854 12S ribosomal RNA gene, partial sequence; mitochondrial	100.000	141	1.75e-65	NA
**L17I6FGUT**	HM563849.1	*Incilius signifer* voucher UTA:A-JRM 4968 tRNA-Phe and 12S ribosomal RNA genes, partial sequence; mitochondrial	100.000	133	4.67e-61	equal hits to other Bufonidae, including Incilius nebulifer
**L16I5GUT**	JN393214.1	*Didelphis virginiana* 12S ribosomal RNA gene, partial sequence	90.071	141	6.42e-50	overlapping signal
**L14I6MEGUT**	ON597633.1	*Homo sapiens* isolate UniPV_126 mitochondrion, complete genome	100.000	137	2.82e-63	NA
**L14I6MGUT**	NA	NA	NA	NA	NA	overlapping signal
**L14I6FGUT**	NA	NA	NA	NA	NA	overlapping signal
**B40I6MGUT**	MG250549.1	*Sus scrofa* isolate LUC208 mitochondrion, complete genome	92.958	142	1.39e-51	overlapping signal
**B40I6FGUT**	ON597633.1	*Homo sapiens* isolate UniPV_126 mitochondrion, complete genome	100.000	137	2.82e-63	NA
**AZ1I6FGUT**	DQ179667.1	*Neotoma albigula* isolate TK74854 12S ribosomal RNA gene, partial sequence; mitochondrial	100.000	141	1.75e-65	NA
**AZ6I6MGUT**	DQ179667.1	*Neotoma albigula* isolate TK74854 12S ribosomal RNA gene, partial sequence; mitochondrial	100.000	141	1.75e-65	NA
**AZ4I5GUT**	DQ179667.1	*Neotoma albigula* isolate TK74854 12S ribosomal RNA gene, partial sequence; mitochondrial	100.000	141	1.75e-65	NA
**AZ1I6FGUT**	DQ179667.1	*Neotoma albigula* isolate TK74854 12S ribosomal RNA gene, partial sequence; mitochondrial	100.000	141	1.75e-65	NA
