# Supplementary material for: Viral metagenome characterization reveals species-specific virome profiles in Triatominae populations from the southern United States
Source: PLoS Negl Trop Dis. 2026 Feb 2;20(2):e0013576. doi: 10.1371/journal.pntd.0013576 (PMC12890172; doi:10.1371/journal.pntd.0013576)
Supplement: S3 Table — List of vOTUs detected in Triatominae natural populations from the southern United States. (PDF) [file pntd.0013576.s003.pdf]

Supplementary Table 3. Viral Operational Taxonomic Units (vOTUs). List of vOTUs detected in Triatominae natural populations from the southern United States.

vOTU	NCBI acc. number	vOTU abbreviation	Species	length (nt)	ORF length (aa)	Protein domains in the sequence (e-value) *	Assembly level	Closest related sequence	Reported host /country	Virus taxon	Baltimore classification
**Triatoma sanguisuga chuviridae vOTU1**	PX139058	Chu_vOTU1	*T. sanguisuga*	15597	2587	RNA-directed RNA polymerase (1.2e-158)	complete cds	Guiyang chuvirus 1 (MZ209784.1)	*Cletus punctiger* / China	*Chuviridae*	ssRNA(-)
					1156	Fusion glycoprotein (0.0063)	complete cds				
					914	RNA virus nucleoprotein (11)	complete cds				
**Triatoma sanguisuga chuviridae vOTU2**	PX139059	Chu_vOTU2	*T. indictiva*	10633	2218	RNA-directed RNA polymerase (1.1e-176)	complete cds	Sanya chuvirus 2 (MZ209828.1)	*Nilaparvata lugens* / China	*Chuviridae*	ssRNA(-)
					471	Envelope glycoprotein (1.5e-27)	complete cds				
					462	RNA virus nucleoprotein (0.027)	complete cds				
**Triatoma indictiva chuviridae vOTU3**	PX139060	Chu_vOTU3	*T. gersteackeri*	1542	513	RNA-dependent RNA polymerase (3.7e-37)	partial cds	Neuropteran chu-related virus OKIAV150 (MW288180.1)	*Eumantispa harmandi* / Japan	*Chuviridae*	ssRNA(-)
**Triatoma gersteackeri chuviridae vOTU4**	PX139061	Chu_vOTU4	*T. gersteackeri*	1539	511	RNA-dependent RNA polymerase (1.1e-94)	partial cds	Sanya chuvirus 2 (MZ209828.1)	*Nilaparvata lugens* / China	*Chuviridae*	ssRNA(-)
**Triatoma gersteackeri chuviridae vOTU5**	PX139062	Chu_vOTU5	*T. gersteackeri*	1505	475	RNA-dependent RNA polymerase (4.7e-20)	partial cds	Sanya chuvirus 2 (MZ209828.1)	*Nilaparvata lugens* / China	*Chuviridae*	ssRNA(-)
**Triatoma indictiva chuviridae vOTU6**	PX139063	Chu_vOTU6	*T. indictiva*	1296	430	RNA-dependent RNA polymerase (2.5e-37)	partial cds	Neuropteran chu-related virus OKIAV150 (MW288180.1)	*Eumantispa harmandi* / Japan	*Chuviridae*	ssRNA(-)
**Triatoma gersteackeri chuviridae vOTU7**	PX139064	Chu_vOTU7	*T. gersteackeri*	858	280	RNA-dependent RNA polymerase (1.9e-40)	partial cds	Blattodean chu-related virus OKIAV148 (MT153417.1)	*Periplaneta americana*/ Germany	*Chuviridae*	ssRNA(-)
**Triatoma gersteackeri virgaviridae vOTU8**	PX139065	Virga_vOTU8	*T. gersteackeri*	9786	2933	Replication proteins (Mtr, Hel and RdRp) (2.7e-66)	complete cds	Xiangshan martelli-like virus 3 (OK491508.1)	Insect / China	*Virgaviridae*	ssRNA(+)
					220	Capsid protein (1.2e-31)	complete cds				
**Triatoma gersteackeri virgaviridae vOTU9**	PX139066	Virga_vOTU9	*T. gersteackeri*	3682	1180	Polyprotein (Mtr, Hel and RdRp) (4e-41)	partial cds	Xiangshan martelli-like virus 2 (OK491507.1)	Insect / China	*Virgaviridae*	ssRNA(+)
**Triatoma gersteackeri virgaviridae vOTU10**	PX139067	Virga_vOTU10	*T. gersteackeri*	2904	702	Polyprotein (Mtr, Hel and RdRp) (3e-67)	partial cds	Xiangshan martelli-like virus 3 (OK491508.1)	Insect / China	*Virgaviridae*	ssRNA(+)
					213	Capsid protein (6.9e-32)	complete cds				
**Triatoma spp. virgaviridae vOTU11**	PX139068	Virga_vOTU11	*T. sanguisuga, T. indictiva*	1227	313	Genome polyprotein (1.8e-41)	partial cds	Pedersore virga-like virus (ON955134.1)	*Ochlerotatus communis* / Finland	*Virgaviridae*	ssRNA(+)
					74	Capsid protein (53)	complete cds				
**Triatoma spp. virgaviridae vOTU12**	PX139069	Virga_vOTU12	*T. sanguisuga, T. indictiva*	1161	214	RNA-dependent RNA polymerase (1.7e-31)	partial cds	Ginka virga-like virus (ON860475.1)	*Ochlerotatus communis* / Sweden	*Virgaviridae*	ssRNA(+)
**Triatoma spp. elliovirales vOTU13R**	PX139070/ PX139071	Ellio_vOTU13	*T. sanguisuga, T. gersteackeri*	7919	2577	Bunyavirus RNA dependent RNA polymerase (4.81e-20)	complete cds	Orius laevigatus bunyavirus 2 (PP908622.1)	*Orius laevigatus* / Spain	Elliovirales	ssRNA(-)
				4670	1065	Envelopment polyprotein (2.5e-73)	complete cds				
**Triatoma spp. elliovirales vOTU14**	PX139072/ PX139073	Ellio_vOTU14	*T. sanguisuga, T. gersteackeri*	7908	2569	Bunyavirus RNA dependent RNA polymerase (1.41e-20)	complete cds	Orius laevigatus bunyavirus 2 (PP908622.1)	*Orius laevigatus* / Spain	Elliovirales	ssRNA(-)
				3499	1058	Glycoprotein (5.8e-79)	partial cds				
**Hospesneotomae protracta arenaviridae vOTU15**	PX139074/ PX139075	Arena_vOTU15	*H. protracta*	7182	2220	Arenavirus RNA polymerase (0e+00), Arenavirus cap snatching domain (3.33e-57)	complete cds	Big brushy tank virus (EU938666.1)	*Neotoma albigula* / USA	*Arenaviridae*	ssRNA(-)
					95	RING finger protein Z (5.9e-53)	complete cds				
				3409	576	Nucleoprotein (8.6e-208), Glycoprotein (4e-148)	partial cds				
**Triatoma rubida arenaviridae vOTU16**	PX139076/ PX139077	Arena_vOTU16	*T. rubida*	1261	236/ 181	RNA-directed RNA polymerase (4.9e-56)	partial cds	Mammarenavirus whitewaterense (NC_010703.1)	*Neotoma albigula* / USA	*Arenaviridae*	ssRNA(-)

**Triatoma indictiva astroviridae vOTU17**	PX139078	Astro_vOTU17	*T. indictiva*	5904	662	Viral protease (0.00075)	partial cds	Flumine Astrovirus 3 (OM954094.1)	River / New Zealand	*Astroviridae*	ssRNA(+)
					546	RNA-dependent RNA polymerase (2.9e-50)	complete cds				
					389	Capsid protein (1.2)	complete cds				
**Hospesneotomae protracta benyviridae vOTU18**	PX139079	Beny_vOTU18	*H. protracta*	5340	1387	Polyprotein (Mtr, Hel and RdRp) (8e-32)	complete cds	Sanya benyvirus 1 (MZ209861.1)	*Sesamia inferens*/ China	*Benyviridae*	ssRNA(+)
					244	Virus coat protein (4e-30)	complete cds				
**Triatoma gersteackeri benyviridae vOTU19**	PX139080	Beny_vOTU19	*T. gersteackeri*	5297	1384	Polyprotein (Mtr, Hel and RdRp) (3.6e-47)	complete cds	Guiyang benyvirus 1 (MZ209815.1)	*Harmonia axyridis*/ China	*Benyviridae*	ssRNA(+)
					253	Virus coat protein (1e-30)	complete cds				
**Triatoma gersteackeri benyviridae vOTU20**	PX139081	Beny_vOTU20	*T. gersteackeri*	5280	1384	Polyprotein (Mtr, Hel and RdRp) (4.2e-48)	complete cds	Benyviridae sp. (PQ792268.1)	*Bradysia coprophila*/ China	*Benyviridae*	ssRNA(+)
					253	Virus coat protein (1.2e-30)	complete cds				
**Hospesneotomae protracta benyviridae vOTU21**	PX139082	Beny_vOTU21	*H. protracta*	2290	667	Replicase polyprotein (Mtr, Hel and RdRp) (7.8e-32)	partial cds	Benyviridae sp. (PQ792268.1)	*Bradysia coprophila*/ China	*Benyviridae*	ssRNA(+)
**Triatoma spp. tombusviridae vOTU22**	PX139083	Tombus_vOTU22	*T. sanguisuga, T. indictiva*	3612	505	RNA-dependent RNA polymerase (1.4e-41)	complete cds	Hemipteran tombus-related virus (QTJ63611.1)	*Notostira elongata* / Germany	*Tombusviridae*	ssRNA(+)
					186	Coat protein (1e-31)	complete cds				
**Triatoma indictiva narnaviridae vOTU23**	PX139084	Narna_vOTU23	*T. indictiva*	2993	921	RNA-directed RNA polymerase (2.6e-21)	complete cds	Wuhan spider virus 7 (NC_033702.1)	Spiders / China	*Narnaviridae*	ssRNA(+)
**Triatoma indictiva narnaviridae vOTU24**	PX139085	Narna_vOTU24	*T. indictiva*	2705	824	RNA-dependent RNA polymerase (5.5e-20)	complete cds	Serbia narna-like virus 3 (MT822185.1)	*Culex pipiens* / Serbia	*Narnaviridae*	ssRNA(+)
**Triatoma indictiva solemoviridae vOTU25**	PX139086	Solemo_vOTU25	*T. indictiva*	2936	501	Protease (6.6e-13)	complete cds	Atrato Sobemo-like virus 2 (MN661089.1)	*Culex* sp / Colombia	*Solemoviridae*	ssRNA(+)
					490	RNA dependent RNA polymerase (7.6e-46)	complete cds				
**Triatoma sanguisuga solemoviridae vOTU26**	PX139087	Solemo_vOTU26	*T. sanguisuga*	2933	562	Peptidase (6.9e-15)	complete cds	Rhodnius prolixus virus 6 (MZ328309.1)	*Rhodnius prolixus* / Brazil	*Solemoviridae*	ssRNA(+)
					506	RNA-dependent RNA polymerase (2.6e-39)	complete cds				
**Triatoma indictiva rhabdoviridae vOTU27**	PX139088	Rhabdo_vOTU27	*T. indictiva*	1856	617	RNA dependent RNA polymerase (5e-108)	partial cds	Sanya conocephalus maculatus rhabdovirus 1 (MZ209844.1)	*Conocephalus maculatus* / China	*Rhabdoviridae*	ssRNA(-)
**Triatoma spp. rhabdoviridae vOTU28**	PX139089	Rhabdo_vOTU28	*T. sanguisuga, T. indictiva*	1273	422	RNA-dependent RNA polymerase (1e-25)	partial cds	Blattodean rhabdo-related virus OKIAV14 (MT153532.1)	*Deropeltis erythrocephala*/ Germany	*Rhabdoviridae*	ssRNA(-)
**Triatoma indictiva partitiviridae vOTU29**	PX139090	Partiti_vOTU29	*T. indictiva*	1800	562	RNA-dependent RNA polymerase (1.9e-36)	partial cds	Hubei partiti-like virus 10 (KX884114.1)	Odonata / China	*Partitiviridae*	dsRNA
**Triatoma spp. partitiviridae vOTU30**	PX139091	Partiti_vOTU30	*T. sanguisuga, T. indictiva*	997	252	RNA-dependent RNA polymerase (7.09e-27)	partial cds	Hangzhou partitivirus 1 (MZ209755.1)	*Orthetrum chrysostigma* / China	*Partitiviridae*	dsRNA
**Triatoma sanguisuga partitiviridae vOTU31**	PX139092	Partiti_vOTU31	*T. sanguisuga*	590	171	RNA-dependent RNA polymerase (7.9e-7)	partial cds	Hubei partiti-like virus 10 (KX884114.1)	Odonata / China	*Partitiviridae*	dsRNA
**Triatominae partitiviridae vOTU32**	PX139093	Partiti_vOTU32	*H. protracta, T. sanguisuga, T. indictiva*	522	167	RNA dependent RNA polymerase (4.7e-19)	partial cds	Hubei partiti-like virus 11 (NC_032139.1)	Odonata / China	*Partitiviridae*	dsRNA
**Triatoma indictiva circoviridae vOTU33**	PX139094	Circo_vOTU33	*T. indictiva*	998	331	Viral coat protein (S domain) (1.27e-08)	partial cds	Circovirus sp. (OP564891.1)	*Neophema chrysogaster* / Australia	*Circoviridae*	ssDNA
**Triatoma spp. circoviridae vOTU34**	PX139095	Circo_vOTU34	*T. rubida, T. sanguisuga, T. indictiva*	992	167	Viral coat protein (S domain) (2.9e-17)	partial cds	Circovirus sp. (OP564891.1)	water / New Zealand	*Circoviridae*	ssDNA
**Triatoma spp. circoviridae vOTU35**	PX139096	Circo_vOTU35	*T. rubida, T. sanguisuga*	568	188	Rep protein (2e-23)	partial cds	Circovirus sp. gt3aAU (PQ754361.1)	*Ixodes ovatus*/ China	*Circoviridae*	ssDNA
**Triatoma spp. circoviridae vOTU36**	PX139097	Circo_vOTU36	*T. rubida, T. indictiva*	507	97	viral replication protein (2.02e-13)	partial cds	Cressdnaviricota sp. (MH616905.1)	red snapper / USA	*Circoviridae*	ssDNA

**Triatoma rubida microviridae vOTU37**	PX139098	Micro_vOTU37	*T. rubida*	734	223	Capsid protein VP1 (3.1e-53)	partial cds	Microvirus sp. (OR349802.1)	*Potamopyrgus antipodarum* / New_Zealand	*Microviridae*	ssDNA
**Triatominae orthomyxoviridae vOTU38**	PX139099	Ortho_vOTU38	*H. protracta, T. indictiva*	516	171	Polymerase PB2 (7.9e-36)	partial cds	Halyomorpha halys orthomyxo-like virus 1 (URQ09140.1)	*Halyomorpha halys*/ Italy	*Orthomyxoviridae*	ssRNA(-)
**Hospesneotomae protracta orthomyxoviridae vOTU39**	PX139100	Ortho_vOTU39	*H. protracta*	354	117	Nucleoprotein (2.2e-17)	partial cds	Hemipteran orthomyxo-related virus OKIAV191 (QMP82198.1)	*Acanthosoma haemorrhoidale*/ Germany	*Orthomyxoviridae*	ssRNA(-)
**Triatoma spp. xinmoviridae vOTU40**	PX139101	Xinmo_vOTU40	*T. rubida, T. gersteackeri*	514	137	RNA dependent RNA polymerase (2e-19)	partial cds	Hangzhou cletus punctiger xinmovirus 1 (UHK03158.1)	*Cletus punctiger*/ China	*Xinmoviridae*	ssRNA(-)
**Triatoma rubida rhabdoviridae vOTU41**	PX139102	Rhabdo_vOTU41	*T. rubida*		278	Mononegavirales RNA dependent RNA polymerase (4e-26)	partial cds	Blattodean rhabdo-related virus OKIAV14 (QMP82336.1)	*Deropeltis erythrocephala*/ Germany	*Rhabdoviridae*	ssRNA(-)
