# Supplementary material for: Viral metagenome characterization reveals species-specific virome profiles in Triatominae populations from the southern United States
Source: PLoS Negl Trop Dis. 2026 Feb 2;20(2):e0013576. doi: 10.1371/journal.pntd.0013576 (PMC12890172; doi:10.1371/journal.pntd.0013576)
Supplement: S4 Table — Summary of raw sequencing reads, reads retained after quality control, total number of assembled contigs, and number of viral reads per sample. (PDF) [file pntd.0013576.s004.pdf]

Supplementary Table 4. Sequencing metrics. Summary of raw sequencing reads, reads retained after quality control, total number of assembled contigs, and number of viral reads per sample.

Individual	Sample name	Raw reads	Reads after QC	Percentage of reads recovered	Assembled contigs per sample	Viral reads per sample	Viral reads %**
AZ1I6F	AZ1I6FGUT	55677990	47555984	85.41253734	226184	3840	0.0080
		55677990	47555984	85.41253734			
	AZ1I6FGONADS	52346468	43235177	82.59425832	230695	14674	0.0339
		52346468	43235177	82.59425832			
AZ6I6M	AZ6I6MGUT	51069430	47880514	93.75572431	304677	14136	0.0295
		51069430	47880514	93.75572431			
	AZ6I6MGONADS	60944909	30347580	49.79510266	261758	8852	0.0291
		60944909	30347580	49.79510266			
B40I6F	B40I6FGUT	45515153	36683575	80.5964005	163413	218014	0.5943
		45515153	36683575	80.5964005			
	B40I6FGONADS	47414182	40834561	86.12309498	235945	523560	1.2821
		47414182	40834561	86.12309498			
B40I6M	B40I6MGUT	51837806	44220414	85.30533487	191623	5116674	11.5708
		51837806	44220414	85.30533487			
	B40I6MGONADS	49369024	28122407	56.96366815	216727	242922	0.8638
		49369024	28122407	56.96366815			
L14I6F	L14I6FGUT	50778094	41929125	82.57325492	230909	116298	0.2773
		50778094	41929125	82.57325492			
	L14I6FGONADS	59722382	49313906	82.57190077	344303	151284	0.3067
		59722382	49313906	82.57190077			
L14I6M	L14I6MGUT	52282492	47226515	90.32950266	266420	2050136	4.341
		52282492	47226515	90.32950266			
	L14I6MGONADS	51084931	37644199	73.68943887	288631	367736	0.9768
		51084931	37644199	73.68943887			
L14I6ME	L14I6MEGUT	51437951	45945068	89.32134175	531140	382602	0.8327
		51437951	45945068	89.32134175			
L16I5	L16I5GONADS	52705047	45453967	86.24215248	350410	461874	1.0161
		52705047	45453967	86.24215248			
	L16I5GUT	51222221	43542511	85.00707339	432263	250274	0.5748
		51222221	43542511	85.00707339			
NM9I6M	NM9I6MGUT	54734683	44614701	81.51084204	199336	328	0.0007
		54734683	44614701	81.51084204			
	NM9I6MGONADS	54631017	35113348	64.2736488	397764	238	0.0006
		54631017	35113348	64.2736488			
L17I6F	L17I6FGUT	48325959	43825313	90.6868977	490561	2744	0.0062
		48325959	43825313	90.6868977			
	L17I6FGONADS	49590199	29780578	60.05335449	268386	3584	0.0001
		49590199	29780578	60.05335449			
AZ4I5	AZ4I5GUT	52525874	42241734	80.42081127	205948	248462	0.5882
		52525874	42241734	80.42081127			
	AZ4I5GONADS	51414803	45067671	87.65504946	330712	231362	0.5134
		51414803	45067671	87.65504946			
AZ1I6F	AZ1I6FABD	47693246	31041027	65.08474386	394736	1316	0.0042
		47693246	31041027	65.08474386			
L19I6F	L19I6FABD	36279160	21725367	59.88387548	186092	28654	0.1319

	36279160	21725367	59.88387548

**The variance observed in viral read proportions likely reflects technical differences between the samples due to varying sequence depths, assembly quality, and total contig yield per sample. While tissue-specific patterns within individual samples could also contribute to this variance, we cannot definitively distinguish biological from technical effects based on the raw read counts of viral reads per sample. However, downstream analyses used normalized read counts per library.
