# Supplementary material for: Viral metagenome characterization reveals species-specific virome profiles in Triatominae populations from the southern United States
Source: PLoS Negl Trop Dis. 2026 Feb 2;20(2):e0013576. doi: 10.1371/journal.pntd.0013576 (PMC12890172; doi:10.1371/journal.pntd.0013576)
Supplement: S5 Table — Contribution of each Viral Operational Taxonomic Units (vOTUs) to the Bray-Curtis dissimilarities observed in Triatominae natural populations between species (H. protracta, T. rubida, T. indictiva, T. sanguisuga, T. gersteackeri). (PDF) [file pntd.0013576.s005.pdf]

Supplementary Table 5. SIMPER analysis. Contribution of each Viral Operational Taxonomic Units (vOTUs) to the Bray-Curtis dissimilarities observed in *Triatoma* natural populations between species (*H.protracta* , *T.rubida, T. indictiva, T.sanguisuga, T.gersteackeri*).

vOTU	Taxon	Av. dissim	Contrib. %	Cumulative %
Solemo_vOTU25	*Solemoviridae*	26.62	26.8	26.8
Solemo_vOTU26	*Solemoviridae*	14.64	14.73	41.54
Ellio_vOTU13	*Elliovirales*	10.61	10.68	52.22
Ellio_vOTU14	*Elliovirales*	7.836	7.888	60.1
Rhabdo_vOTU41	*Rhabdoviridae*	7.79	7.842	67.95
Narna_vOTU24	*Narnaviridae*	6.302	6.344	74.29
Arena_vOTU15	*Arenaviridae*	4.64	4.671	78.96
Beny_vOTU19	*Benyviridae*	3.019	3.039	82
Xinmo_vOTU40	*Xinmoviridae*	2.332	2.347	84.35
Virga_vOTU8	*Virgaviridae*	2.066	2.08	86.43
Chu_vOTU3	*Chuviridae*	1.6	1.611	88.04
Ortho_vOTU38	*Orthomyxoviridae*	1.213	1.221	89.26
Chu_vOTU4	*Chuviridae*	1.143	1.15	90.41
Ortho_vOTU39	*Orthomyxoviridae*	1.068	1.075	91.48
Virga_vOTU10	*Virgaviridae*	1.054	1.061	92.54
Chu_vOTU7	*Chuviridae*	1.05	1.057	93.6
Chu_vOTU5	*Chuviridae*	1.033	1.04	94.64
Beny_vOTU18	*Benyviridae*	0.9424	0.9487	95.59
Virga_vOTU9	*Xinmoviridae*	0.9375	0.9438	96.53
Circo_vOTU35	*Circoviridae*	0.7596	0.7647	97.3
Arena_vOTU16	*Arenaviridae*	0.5447	0.5484	97.85
Partiti_vOTU32	*Partitiviridae*	0.3718	0.3743	98.22
Circo_vOTU34	*Circoviridae*	0.341	0.3433	98.56
Micro_vOTU37	*Microviridae*	0.2801	0.282	98.85
Chu_vOTU2	*Chuviridae*	0.2587	0.2605	99.11
Virga_vOTU12	*Virgaviridae*	0.1995	0.2009	99.31
Circo_vOTU36	*Circoviridae*	0.1522	0.1532	99.46
Virga_vOTU11	*Virgaviridae*	0.1185	0.1193	99.58
Tombus_vOTU22	*Tombusviridae*	0.1089	0.1097	99.69
Partiti_vOTU31	*Partitiviridae*	0.07963	0.08016	99.77
Beny_vOTU20	*Benyviridae*	0.07289	0.07338	99.84
Astro_vOTU17	*Astroviridae*	0.06069	0.06109	99.9
Beny_vOTU21	*Benyviridae*	0.03512	0.03535	99.94
Partiti_vOTU30	*Partitiviridae*	0.03346	0.03368	99.97
Narna_vOTU23	*Narnaviridae*	0.009685	0.00975	99.98
Rhabdo_vOTU29	*Rhabdoviridae*	0.007088	0.007136	99.99
Chu_vOTU1	*Chuviridae*	0.005412	0.005448	100
Chu_vOTU6	*Circoviridae*	0.002119	0.002133	100
Circo_vOTU33	*Circoviridae*	0.001271	0.00128	100
Rhabdo_vOTU27	*Rhabdoviridae*	0.000618	0.0006222	100
Rhabdo_vOTU28	*Rhabdoviridae*	0.000252	0.0002537	100
