# Supplementary figures and images for: Viral metagenome characterization reveals species-specific virome profiles in Triatominae populations from the southern United States
Source: PLoS Negl Trop Dis. 2026 Feb 2;20(2):e0013576. doi: 10.1371/journal.pntd.0013576 (PMC12890172; doi:10.1371/journal.pntd.0013576)

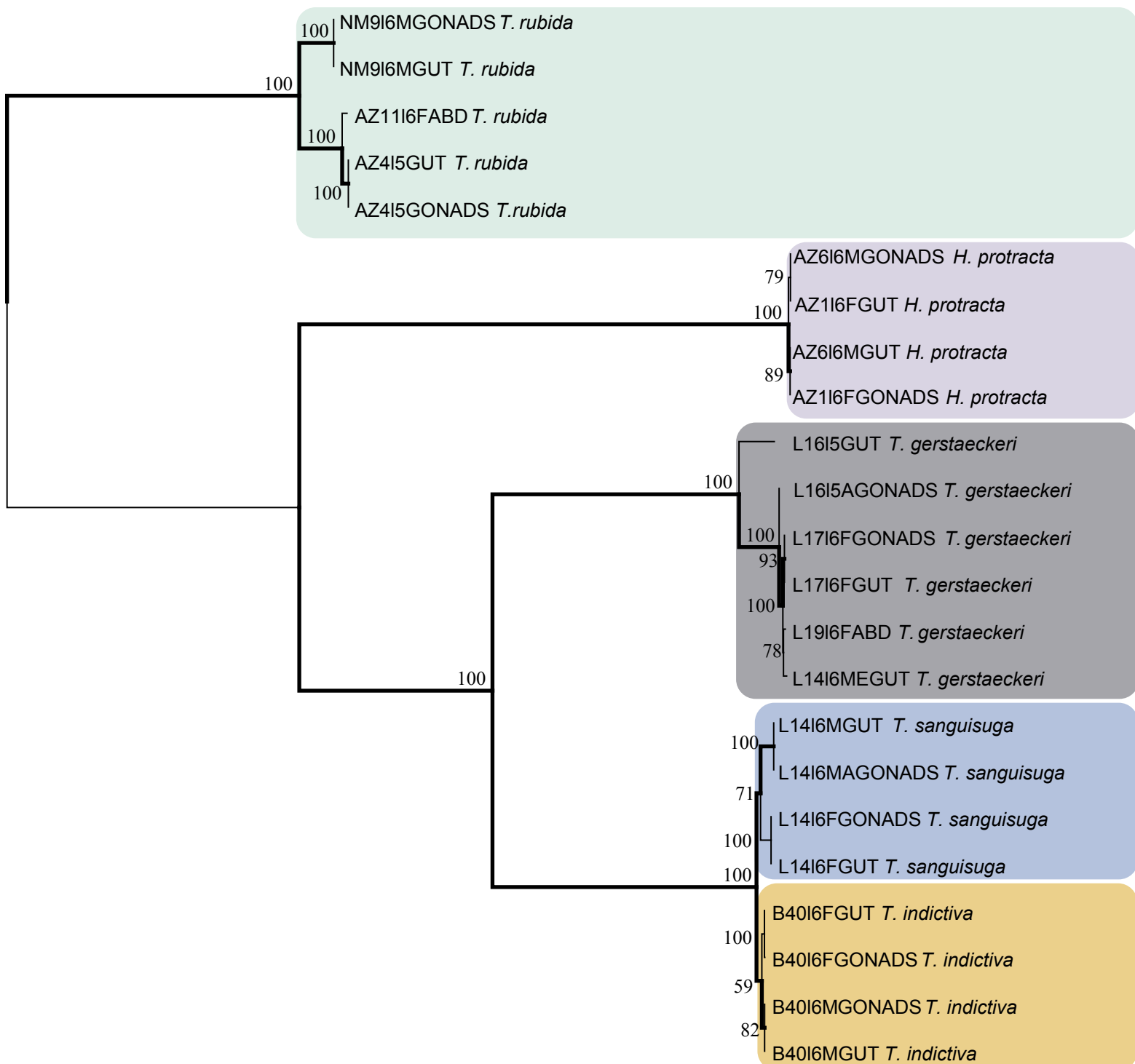

0.04

Supplement: S1 Fig — Maximum-likelihood tree based on eight mitochondrial genes retrieved from each tissue sample analyzed in this study which correspond to five species (Triatoma rubida, Hospesneotomae protracta, Triatoma gerstaeckeri, Triatoma sanguisuga, and Triatoma indictiva). Multiple libraries from the same individual are shown as an internal quality-control measure to confirm consistent mitochondrial signal and detect potential issues such as sample mislabeling or multiplexing errors. Node labels indicate bootstrap support values (100 replicates). (PDF) [file pntd.0013576.s006.pdf]
