# Supplementary material for: Viral metagenome characterization reveals species-specific virome profiles in Triatominae populations from the southern United States
Source: PLoS Negl Trop Dis. 2026 Feb 2;20(2):e0013576. doi: 10.1371/journal.pntd.0013576 (PMC12890172; doi:10.1371/journal.pntd.0013576)
Supplement: S2 Fig — A) Non-metric multidimensional scaling (NMDS) based on Bray-Curtis distances, showing clustering patterns by study location (PERMANOVA, p = 0.003; R² = 0.171), sex (PERMANOVA, p = 0.426; R² = 0.093), and tissue (PERMANOVA, p = 0.97; R² = 0.138). B) Shannon diversity index of vOTUs by location (Kruskal-Wallis test, p = 0.816), sex (Kruskal-Wallis test, p = 0.320), and tissue (Kruskal-Wallis test, p = 0.744). C) Venn diagrams displaying the number of shared vOTUs among triatomine populations, grouped by location, sex, and tissue type. (PDF) [file pntd.0013576.s007.pdf]

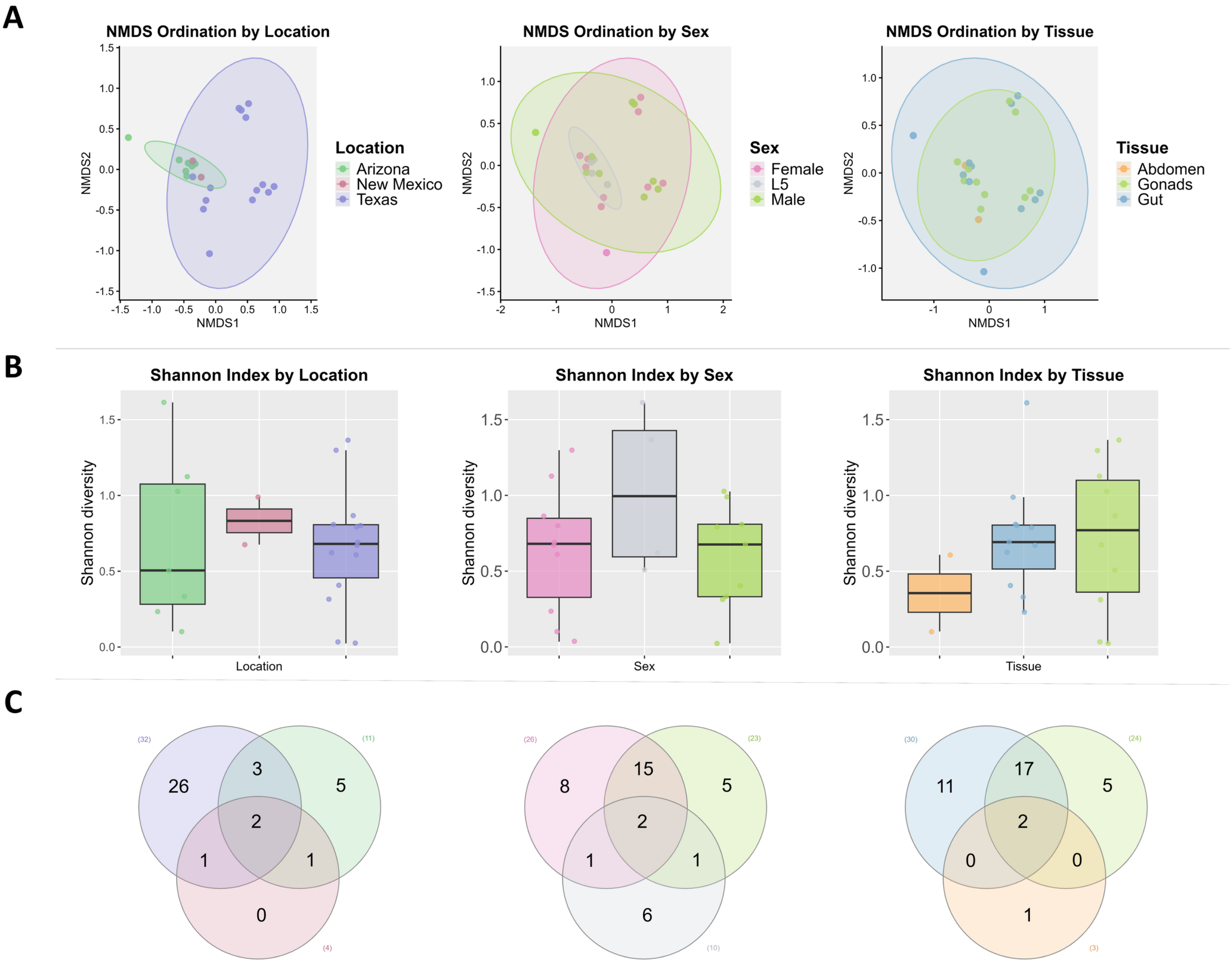

Supplementary Figure 2. Alpha and beta diversity of vOTUs in natural populations of triatomines. A) Non-metric multidimensional scaling (NMDS) based on Bray-Curtis distances, showing clustering patterns by study location (PERMANOVA, $p = 0.003$; $R^2 = 0.171$), sex (PERMANOVA, $p = 0.426$; $R^2 = 0.093$), and tissue (PERMANOVA, $p = 0.97$; $R^2 = 0.138$). **B)** Shannon diversity index of vOTUs by location (Kruskal-Wallis test, $p = 0.816$), sex (Kruskal-Wallis test, $p = 0.320$), and tissue (Kruskal-Wallis test, $p = 0.744$). **C)** Venn diagrams displaying the number of shared vOTUs among *Triatoma* populations, grouped by location, sex, and tissue type.
