# Supplementary material for: Viral metagenome characterization reveals species-specific virome profiles in Triatominae populations from the southern United States
Source: PLoS Negl Trop Dis. 2026 Feb 2;20(2):e0013576. doi: 10.1371/journal.pntd.0013576 (PMC12890172; doi:10.1371/journal.pntd.0013576)
Supplement: Attachment — Submitted filename: ReviewersComments.docx [file pntd.0013576.s009.docx]

Dear Editors and Reviewers,
below, we provide our responses (***bold italics***) to all comments. Line numbers correspond to the revised manuscript with tracked changes.

**Journal Requirements:**

1) Please provide an Author Summary. This should appear in your manuscript between the Abstract (if applicable) and the Introduction, and should be 150-200 words long. The aim should be to make your findings accessible to a wide audience that includes both scientists and non-scientists. Sample summaries can be found on our website under Submission Guidelines:
https://journals.plos.org/plosntds/s/submission-guidelines#loc-parts-of-a-submission

***A concise Author Summary has been added within the above-mentioned sections (Lines 35-48).***

2) Some material included in your submission may be **copyrighted**. According to PLOSu2019s copyright policy, authors who use figures or other material (e.g., graphics, clipart, maps) from another author or copyright holder must demonstrate or obtain permission to publish this material under the Creative Commons Attribution 4.0 International (CC BY 4.0) License used by PLOS journals. Please closely review the details of PLOSu2019s copyright requirements here: PLOS Licenses and Copyright. If you need to request permissions from a copyright holder, you may use PLOS's Copyright Content Permission form.
**Please respond directly to this email and provide any known details concerning your material's license terms and permissions required for reuse**, even if you have not yet obtained copyright permissions or are unsure of your material's copyright compatibility. Once you have responded and addressed all other outstanding technical requirements, you may resubmit your manuscript within Editorial Manager. 
Potential Copyright Issues:- Figure 1. Please (**a**) **provide a direct link to the base layer** of the map (i.e., the country or region border shape) and ensure this is also included in the figure legend; and (**b) provide a link to the terms of use / license information for the base layer image or shapefile. We cannot publish proprietary or copyrighted maps (e.g. Google Maps, Mapquest) and the terms of use for your map base layer must be compatible with our CC BY 4.0 license.**
Note: if you created the map in a software program like R or ArcGIS, please locate and indicate the source of the basemap shapefile onto which data has been plotted.
If your map was obtained from a copyrighted source please amend the figure so that the base map used is from an openly available source. Alternatively, please provide explicit written permission from the copyright holder granting you the right to publish the material under our CC BY 4.0 license.
If you are unsure whether you can use a map or not, please do reach out and we will be able to help you. The following websites are good examples of where you can source open access or public domain maps:
* U.S. Geological Survey (USGS) - All maps are in the public domain. (http://www.usgs.gov)
* PlaniGlobe - All maps are published under a Creative Commons license so please cite u201cPlaniGlobe, http://www.planiglobe.com, CC BY 2.0u201d in the image credit after the caption. (http://www.planiglobe.com/?lang=enl)
* Natural Earth - All maps are public domain. (<http://www.naturalearthdata.com/about/terms-of-use/>).

***The map in Figure 1 includes two layers: the Base Map and the Ecoregions. Both are under public domain.***

- ***Base map: U.S. administrative boundaries from geoBoundaries (ADM1). Public domain.***

***Source:*** [***https://www.geoboundaries.org***](https://www.geoboundaries.org/)

- ***Ecoregions: NA_CEC_Eco_Level1 (U.S. EPA). Public domain.***

***Source:*** [***https://www.epa.gov/eco-research/ecoregions-north-america***](https://www.epa.gov/eco-research/ecoregions-north-america)

***License info:*** [***https://edg.epa.gov/epa_data_license.html***](https://edg.epa.gov/epa_data_license.html)

***Additionally, we have added the link to the layers used at the end of Figure 1 caption.***

3) Please amend your detailed Financial Disclosure statement. This is published with the article. It must therefore be completed in full sentences and contain the exact wording you wish to be published.
- State the initials, alongside each funding source, of each author to receive each grant. For example: "This work was supported by the National Institutes of Health (####### to AM; ###### to CJ) and the National Science Foundation (###### to AM)."
- State what role the funders took in the study. If the funders had no role in your study, please state: "The funders had no role in study design, data collection and analysis, decision to publish, or preparation of the manuscript.".
If you did not receive any funding for this study, please simply state: u201cThe authors received no specific funding for this work.u201d

***Financial Disclosure statement has been added (Lines 549-552).***

**Reviewers' Comments:**

Reviewer's Responses to Questions

**Key Review Criteria Required for Acceptance?**


As you describe the new analyses required for acceptance, please consider the following:


**Methods**


-Are the objectives of the study clearly articulated with a clear testable hypothesis stated?

-Is the study design appropriate to address the stated objectives?

-Is the population clearly described and appropriate for the hypothesis being tested?

-Is the sample size sufficient to ensure adequate power to address the hypothesis being tested?

-Were correct statistical analysis used to support conclusions?

-Are there concerns about ethical or regulatory requirements being met?

**Reviewer #1:** The objectives of the study are clearly stated, and the study addresses an important knowledge gap in Triatominae virome characterization. However, the sampling design requires clarification. The manuscript sometimes uses “sample” to denote sequencing libraries and other times to denote individuals (insects), which complicates interpretation. Inconsistent suffix labeling (e.g., “gonads,” “ovary,” “Agonad,” “BOVA,” “testes,” “abdomen,” “BGUT,” “gut,” “ovaries”) of tissues across figures and supplementary materials further obscures whether multiple libraries from the same insect were treated as independent observations.

***We thank the reviewer for pointing this out. We have made all the labeling consistent across the manuscript, figures and supplementary materials. We have extended the text of the Sample Set section to provide more detailed explanation on used samples (Lines 99-110).***

If so, this compromises assumptions of independence in statistical tests and should either be avoided or acknowledged. The number of insects included is mentioned, but the treatment of multiple tissue types per insect in diversity analyses is not explicit. This ambiguity weakens confidence in the robustness of the statistical framework. I recommend the authors clarify definitions of “sample” and “triatomine individual,” specify the analytical unit used for diversity analyses, and consider alternative approaches (e.g., aggregating libraries per individual or treating tissue as a dependent variable).Considering the large number of variables included (triatomine species, area, predominant host species, stage/sex, tissue type, and T. cruzi infection status) in relation to the small sample size (23 libraries derived from tissues of approximately 11–16 triatomine individuals), the statistical power is limited.

***We are aware of the limitations caused by the small sample size and have been cautious in analyzing and interpreting the results. Despite these limitations, we consider that the data we collected and analyzed provide important insights into viral composition in triatomines, especially given the scarcity of virome studies in these insects.***

***We included 13 individuals in this study, from which 23 tissue samples were used for separate sequencing libraries. We analyzed tissue sample derived data separately to obtain a more detailed view of viral abundance and diversity, which can differ substantially among tissues. The analytical unit for alpha diversity and beta diversity was the tissue sample. The diversity analyses using the metatranscriptomic data follow standard practices in metavirome studies, as noted in the manuscript. Although we recognize that this design is exploratory and carries limitations, it provides a more detailed characterization of viral community dynamics within hosts.***

***All statistical analyses were performed using standard assumptions and properly accounted for the relationship between specimens and their corresponding tissues. The results presented in the manuscript correspond to the analysis of the 23 tissue samples, which show a clear grouping by species in the ordination analyses. For this reason, the reported statistics treat the 23 libraries as independent units. To address the reviewer's concern and evaluate the effect of treating tissues separately, we repeated the analyses using aggregated normalized abundances at the individual level. The results are consistent with those obtained using the tissue-level dataset: Triatominae species (PERMANOVA p < 0.001, R² = 0.59657), Location (PERMANOVA p = 0.023, R² = 0.23404), and Sex (PERMANOVA p = 0.956, R² = 0.11753). These outcomes indicate that treating the tissues as independent samples did not affect the overall patterns detected in our study (these analyses were conducted only to address the reviewer concern and the statistical results shown in the manuscript are the ones included initially).***

***In response to the reviewer’s comment, we also explicitly acknowledge the limitations in the revised discussion section, noting that the sample size may limit the generalization of the results. We have modified the paragraph at the end of the discussion as follows:***

***Lines 522-531:*** *‘In addition to challenges related to EVEs, our study is constrained by the relatively small sample size, which may limit the generalizability of our findings, particularly regarding the effects of geographic variation on viral abundance and composition in triatomine natural populations. Overcoming these limitations will require expanded sampling efforts together with complementary approaches, such as whole-genome and small RNA sequencing, to assess viral replication and host antiviral responses (65, 73). Integrating these methodologies in future work will improve the distinction between EVEs and actively replicating viruses in Triatominae and provide deeper insight into virus–host interactions. Moreover, broader sampling across the geographic and ecological ranges of triatomine species will be essential for clarifying the factors that shape viral diversity in these important disease vectors.’*

Blood meal identification was performed using Sanger sequencing of 12S fragments, which typically detects only the dominant host. Notably, overlapping host signals observed in Supplementary Table S2 suggest the presence of mixed templates, and recent metabarcoding studies have shown that multiple blood meals are frequent in triatomines. This methodological limitation should be acknowledged in the discussion. As illustrated in Figure 5, the clustering patterns and vOTU distributions should therefore be interpreted with caution, reinforcing that these results are exploratory rather than conclusive.

***To clarify this point, we have modified the related methodology and result sections to specify that we identified the dominant bloodmeal as follows. Line 120:*** ‘*To identify the dominant bloodmeal source, analysis was conducted with 12 DNA isolates derived from gut tissues...’* ***Line 223:*** *"For ten Triatominae individuals, dominant blood meal analysis identified five different hosts...*". ***However, this approach does not anyhow change the interpretation of results presented in Figure 5.***

It is not entirely clear how rRNA removal was performed prior to downstream analyses. While the results are reported as “after removal of rRNA” (Ln. 195), the methods section does not specify whether this step was carried out experimentally (e.g., using an rRNA depletion kit during library preparation) or computationally (bioinformatic filtering of rRNA reads).

***The rRNA was depleted during the library preparation and an additional removal step was carried out computationally. These methodological details have been added to the manuscript (Lines 118 and 129-131).***

At Ln. 455–465, the authors report that beta diversity analyses revealed species-specific clustering patterns, while sex and tissue type had minimal influence. However, it is not clear what the unit of analysis was for beta diversity — whether the analyses were conducted at the level of the 23 nucleic acid libraries, aggregated by individual insect, or directly by species. Explicitly stating the analytical unit would improve transparency and allow readers to better evaluate the robustness of the diversity patterns reported.

***As explained above, we have clarified throughout the manuscript when referring to the 13 specimens used in the study or to the 23 tissue samples used for analysis. For clarity, we have added an additional line to the methodology section regarding diversity analysis, as follows:***

***Lines 193-194***: ‘*Diversity analyses were conducted with an abundance-based matrix derived from tissue samples...’*

No major ethical or regulatory concerns were identified.

Reviewer #2: The hypothesis and objectives of the study are clearly stated and articulated, also well developed step by step in the methodology section. The population is clearly described, altought the number of samples is small. The samples info is detailed in Table S1, but it would be appreciated if you could add a few words in Sample set section where you describe how many specimens you collected per each sampling state, and also explain why you selected those 2 females for whole abdomen. The statistical analysis is fine for a good approximation to test the hypothesis.

***The Sample Set section was extended to provide a detailed information (Lines 100-110).***

**Results**


-Does the analysis presented match the analysis plan?

-Are the results clearly and completely presented?

-Are the figures (Tables, Images) of sufficient quality for clarity?

Reviewer #1: The results are presented in detail, but some issues require clarification:

References to figures are occasionally confusing. For example, **Ln. 393–400 describes viral diversity but cites Supplementary Figure S1**, which actually shows mitochondrial triatomine phylogeny. This must be corrected.

***The correct Supplementary figure has been cited in the manuscript.***

In Supplementary Figure S1, multiple libraries from the same insect appear as independent COI terminals, which does not provide additional phylogenetic information and risks confusing readers. Either one representative per insect should be shown, or the rationale should be justified.

***We agree that this does not add phylogenetic information and now clarify in the figure caption that the phylogenetic analysis based on 8 mitochondrial genes assembled independently from each library served as an internal quality-control standard. Including all libraries from the same individual allows us to verify the consistency of their mitochondrial signal and to detect potential issues such as sample mislabeling, cross-contamination, or multiplexing errors.***

In Supplementary Figure S2, beta diversity analyses are reported by location, but species identity and location are not independent. For instance, T. rubida was collected in both Arizona and New Mexico, while other species were restricted to single sites. This collinearity complicates interpretation, and the authors should clarify whether clustering reflects species, geography, or both.

***We agree that species and geography are not fully independent in our sampling design and the metavirome structure should be interpreted with caution. We originally acknowledged the possible interplay between geography and species specificity at both the results (Lines 382-394) and discussion section (Lines 503 – 514). However, the clustering analyses (Figure 5 and Supplementary Figure 2A) demonstrate that virome composition is primarily structured by host species. Notably,* T. rubida *individuals from both Arizona and New Mexico cluster together, indicating species-level consistency despite geographic separation. Species restricted to Texas also cluster distinctly from one another, further supporting species-specific virome signatures. We have adjusted Figure 5A to show both geographical and species origin of the clustered samples. Additionally, at the end of the discussion we underscore that the small sample size constrains our ability to derive robust conclusions regarding the influence of geographic variation on viral composition in these triatomine populations.***

Supplementary Table S4 shows large variance in viral read proportions (<0.001% to >5%), which deserves explicit discussion as it may reflect both biology and technical bias.

***We thank the reviewer for pointing his out. The large variance was due to calculations considering both the length of the viral contigs and their coverage. Assembly fragmentation then affected the calculated proportion. We have now opted for a simple calculation of viral read proportions obtained by mapping identified viral sequences back to the raw read libraries. For individual samples, viral read percentages were computed as (viral reads / total reads) × 100. The text has been adjusted as follows: “****Sequencing of 23 Triatominae tissue samples generated a total read length of 277 Gb. Following quality control, the reads were de novo assembled into 6,748,633 contigs. Viral RNA constituted a mean of 0.307% of the total RNA per sample (post rRNA removal), with an interquartile range (IQR) of 0.019%-0.848%.****” Supplementary Table 4 was updated. The observed variance is still considerable and likely reflects technical differences between samples. Each sample represents an independent library with varying sequence depths, assembly quality and total contig yield, which affects viral sequence detection and quantification. However, tissue-specific patterns within individual samples may reflect biological differences, we cannot confirm the viral read differences in different samples due to these technical limitations solely based on these raw values. Nonetheless, the downstream analysis in the study was conducted by normalizing the number of reads per library to avoid any technical bias. Given the points for the above, we now added a clarification as a footnote to the respective table.***

Overall, figures and tables are of sufficient quality but would benefit from more standardized labeling (e.g., tissues named consistently as “gut,” “gonads,” “ovaries,” etc.), as previously said.

***The labeling has been unified.***

Reviewer #2: The section present the results clearly and it was a wise decision to divide the results at first according to the viruses category, it makes the reading easier.
The figures are clear, but (after download) they loose resolution and become pixelated when zoomed in. If possible, please consider providing higher-resolution versions to ensure readability.

***We have provided higher resolution figures.***

The tables are complete, detailed and the information is well organized.
Figure S2 presents important data that supports part of the objectives of the study (section "Geographic specificity of the Triatominae metavirome"). Therefore, I recommend including Figure S2 in the main manuscript, considering it represents graphically the indices used to test the hypothesis, and they are well arranged to enhance the understanding of these results.

***We appreciate the reviewer for recommending the inclusion of Figure S2 in the main manuscript. However, we opted to include the geographical origin into Figure 5, which now better highlights the species-specific pattern observed in our study.***

In line 393, T. cruzi is not in italics.

***This has been corrected.***

Regarding the discussion section:
Line 409 and 410: I recommend a little change: "(...) viral composition and abundance COULD BE/MAY BE primarily shaped (...)", as you described later in the discussion (line 471-473) that your findings SUGGEST (...) and (...) MAY primarily be shaped by species identity". This just to avoid overgeneralizations, considering that the dataset included a limited number of species and few specimens per species.

***We thank the reviewer for pointing this out. The text has been adjusted to the discussion section as follows: Lines 439-441: “****Our findings suggest that viral composition and abundance are primarily shaped by Triatominae species identity, highlighting a species-specific viral metagenome profile****”.***

Also, please check the scientific names in italics since line 418, and in the whole document just in case.

***This formatting issue has now been fully corrected.***

Line 429-431 --> to this statement: "While our study does not assess whether triatomines contribute to plant virus transmission, our findings highlight a potential ecological role warranting further investigation", maybe you can complement with information regarding herbivory in Triatomines and the ecological implications, it could contribute to improve this part of your discussion and deepen a little further to help explain your findings, for example please consider these papers : https://doi.org/10.1186/s13071-016-1401-0 ; https://doi.org/10.1101/2023.03.03.530934 ; https://doi.org/10.1590/s0074-02762011000400016

***We have extended the discussion and acknowledged the recommended papers (lines 460-466).***

**Conclusions**


-Are the conclusions supported by the data presented?

-Are the limitations of analysis clearly described?

-Do the authors discuss how these data can be helpful to advance our understanding of the topic under study?

-Is public health relevance addressed?

Reviewer #1: The conclusions are generally consistent with the data but should be presented more cautiously. With 23 libraries derived from ~11–16 insects, statistical power is limited, and treating multiple tissue libraries as independent units may bias results. The discussion should explicitly acknowledge these limitations. The findings are relevant to understanding viruses diversity in hematophagous vectors, but the impact would be strengthened by clarifying whether species identity or geography was the main driver of beta diversity.

***As previously noted, based on the reviewer recommendations, we have revised the manuscript to clarify how the analyses were carried out and to carefully discuss the results concerning the impact of species and geographic factors on the structure of the studied metaviromes. In the final paragraph of the discussion, we also acknowledged the main limitations of our study, including the sample size and the challenge of making definitive conclusions about the influence of geographic factors in our study.***

Reviewer #2: I suggest: "(...)the most comprehesive characterization of Triatominae virome to date IN THE US".
Please state some limitations in the conclusion as well.
The conclusion is clear and detail the main findings that make the study relevant for public health.

***The conclusion text has been revised accordingly.***

**Editorial and Data Presentation Modifications?**


Use this section for editorial suggestions as well as relatively minor modifications of existing data that would enhance clarity. If the only modifications needed are minor and/or editorial, you may wish to recommend “Minor Revision” or “Accept”.

Reviewer #1:

-Standardize terminology for tissues across figures, tables, and supplementary material.

***Has been standardized.***

-Revise figure legends to avoid misleading terminology (e.g., “Host phylogeny of sampled Triatominae species.” > “phylogenetic reconstruction of Triatominae species”). The term host may be misleading, as it is generally used to refer to vertebrate blood meal sources rather than insect vectors.

***We agree with the reviewer. The term host has been either specified further or replaced.***

-Ensure consistent use of “sample” (=libraries?) in relation to “triatomine bugs”

***This has been corrected.***

Reviewer #2: Check the scientific names, some are not in italics, and, if possible, enhance the figures quality

***Have been revised.***

**Summary and General Comments**


Use this section to provide overall comments, discuss strengths/weaknesses of the study, novelty, significance, general execution and scholarship. You may also include additional comments for the author, including concerns about dual publication, research ethics, or publication ethics. If requesting major revision, please articulate the new experiments that are needed.

Reviewer #1: This is an original and valuable contribution, representing a considerable investment of effort and resources. The authors assembled viral contigs from Triatominae nucleic acid libraries, recovering a broad diversity of ssRNA(+), ssRNA(–), dsRNA, and ssDNA viruses and exploring their evolutionary relationships. This is novel and relevant for PLOS NTD. However, several issues limit interpretability: (i) lack of clarity in distinguishing “samples” from “triatomine individuals,” (ii) inconsistent nomenclature of tissue libraries, (iii) unclear statistical treatment of multiple libraries from the same insect, and (iv) potential collinearity between species and location in beta diversity analyses. Addressing these points would strengthen both methodological rigor and transparency. I therefore recommend Major Revision, with emphasis on clarifying sampling design, analytical units, and limitations of independence in statistical tests.

Reviewer #2: It is a well-designed an carefully executed study with meaningful findings for the field and public health. Although the sample size is small, it provides a solid and valuable basis for future research on this topic.

***We hope we have thoroughly addressed all the issues above.***
